# Regulatory T Cells and the Control of the Allergic Response

**DOI:** 10.1155/2012/948901

**Published:** 2012-09-29

**Authors:** Ana Agua-Doce, Luis Graca

**Affiliations:** ^1^Instituto de Medicina Molecular, Cellular Immunology Unit, Faculdade de Medicina da Universidade de Lisboa, Avenida Professor Egas Moniz, 1649-028 Lisbon, Portugal; ^2^Instituto Gulbenkian de Ciência, Cellular Immunology Unit, 2781-901 Oeiras, Portugal

## Abstract

The study of immune regulation and tolerance has been traditionally associated with self/nonself-discrimination. However, the finding that dominant tolerance, a model that puts in evidence the active role of regulatory T cells, can develop to nonself-antigens suggests that the imposition of tolerance can be context dependent. This paper reviews the emerging field of acquired immune tolerance to non-self antigens, with an emphasis on the different subsets of induced regulatory T cells that appear to specialize in specific functional niches. Such regulatory mechanisms are important in preventing the onset of allergic diseases in healthy individuals. In addition, it may be possible to take advantage of these immune regulatory mechanisms for the induction of tolerance in cases where pathological immune responses are generated to allergens occurring in nature, but also to other immunogens such as biological drugs developed for medical therapies.

## 1. Introduction

For many decades the self/nonself-discrimination by the immune system was assumed to be a consequence of clonal selection of effector T cells. Compelling evidence has, however, imposed a revised view of self/nonself-discrimination: dominant regulatory mechanisms, where regulatory T (Treg) cells play a central role, are essential for maintenance of self-tolerance [1]. But recently it is becoming apparent that the importance of dominant regulation goes beyond the discrimination of self and nonself: it also discriminates between harmful and innocuous. In fact, cellular mechanisms, as detailed below, persistently patrol the organism preventing the onset of inflammation, namely, allergic inflammation. The biological significance of this active tolerance-imposing mechanism is well demonstrated by the severity of the allergic and autoimmune syndrome that arises in individuals that lack these ability to tolerate self- and harmless antigens. 

Indeed, the organism is constantly exposed to nonpathogenic antigens that, in healthy individuals, are tolerated. It is, however, common (and becoming increasingly frequent) that an overzealous immune system will activate and develop effector responses to such harmless antigens developing allergy and other inflammatory diseases. Over the last decades allergic diseases, including allergic asthma, atopic dermatitis, and food allergy, have become a major health problem in developed countries [2]. Despite the advances in the understanding of the pathophysiology of allergy and in its clinical management, allergic pathology remains a significant burden on the quality of life and economy of western society. Several strategies have been devised to overcome the pathological immune response by inducing immune tolerance. This paper reviews the impact of dominant regulatory mechanisms in the maintenance of tolerance to foreign antigens, including allergens.

A major cellular mechanism in maintaining immune tolerance is the population of natural (or thymic-derived) Foxp3^+^ Treg cells [3, 4]. Indeed these have been clearly implicated as potent inducers of a nonresponsive state in several immune-mediated pathologies like autoimmunity, transplantation, graft-versus-host disease, and allergy [5–9]. It has been shown, in allergy, that regulatory T cells can be transferred conferring specific tolerance to subsequent challenges with the allergen [10, 11]. In addition, depletion of the regulatory T cells can have a detrimental effect in allergic airway hyperreactivity [12]. Importantly Foxp3 deficiency, in mice and human beings, leads to a severe immune disregulation syndrome characterized by allergic and autoimmune manifestations that are rapidly fatal [13]. In addition to the important role of natural Foxp3^+^ Treg cells (nTreg) in preventing autoimmunity, it has become established that Foxp3 expression can be peripherally induced following T-cell activation in presence of TGF-*β* [14]. These peripherally induced Treg cells (iTreg) are believed to be important for tolerance induction to nonselfantigens, including allergens [14]. 

## 2. Induction of Regulatory T Cells

The study of peripheral induction of Treg cells was greatly facilitated with the use of *Rag*-insufficient TCR-transgenic mice, with the TCR specific for a nonselfantigen. In these mice nTregs cannot be formed in the thymus due to the absence of a selecting thymic antigen. In 2003 it was shown that conventional T cells can be converted into iTreg *in vitro* when activated in presence of TGF-*β* [15]. In addition those iTreg cells were fully capable of controlling airway hyperreactivity (AHR) in previously sensitized mice [15–19]. It was subsequently found that reducing or blocking the available amount of TGF-*β* exacerbates AHR [20, 21], while the local delivery of this cytokine or adoptive transfer of T cells engineered to express latent TGF-*β* rescue mice from antigen sensitization and therefore prevent AHR [22, 23]. Interestingly, suboptimal TCR signaling together with TGF-*β* greatly enhances iTreg conversion [24], which is in agreement with *in vivo* data showing that repeated low doses of allergen exposure promotes the emergence of Foxp3^+^ iTregs expressing TGF-*β* on the membrane [25]. Under sub-optimal TCR stimulation, which can be obtained by using a low dose of plate bound anti-CD3 or DCs pulsed with a low dose of agonist peptide or with downmodulation of the TCR with nondepleting anti-CD4, iTreg conversion is promoted in the absence of exogenous TGF-*β* [26]. Under those conditions Foxp3 expression still requires TGF-*β*, but the T cells can produce TGF-*β* and benefit from the presence of this cytokine for conversion to Treg [26]. 

In addition to the importance of TGF-*β* for iTreg conversion, some studies showed that TGF-*β* can directly inhibit GATA3 expression thus impairing Th2 differentiation [27–29]. Because the Th2 response is impaired, the production of IL-4 is diminished, and this has a direct impact on B-cell class switch preventing IgE and favoring IgA production [30].

It is also becoming apparent that the environment influences the outcome of T-cell activation and the decision to induce Foxp3 and regulatory properties. Several reports have shown that the mucosal surfaces have a role in establishing an iTreg population: alveolar epithelial cells have been reported to participate in iTreg induction in a mechanism dependent of MHC class II expression and TGF-*β* [31]. Both alveolar and gut epithelia have been shown to depend on retinoic acid together with TGF-*β* to induce tolerance [32, 33]. It was also found that retinoic acid in the presence of TGF-*β* impaired STAT6 binding to the Foxp3 promoter therefore enhancing histone acetylation and reverting the repressive effect of IL-4 on the Foxp3 promoter [34]. 

Despite the critical role of TGF-*β* in iTreg induction and henceforth tolerance, this cytokine can also have some adverse effects since it is instrumental in the differentiation of Th17 and Th9 together with IL-6 and IL-4, respectively [35, 36]. Note that although Treg cells can prevent allergic autoimmune encephalomyelitis (EAE), mice with T cells with a dominant negative receptor for TGF-*β*1 do not develop EAE as Th17 cells are not induced [37]. Moreover, TGF-*β* has also been implicated in tissue remodeling, by induction of collagen expression in fibroblasts, as well as goblet cell proliferation and mucus production [38]. 

## 3. Regulatory T Cells and IL-10

Although TGF-*β* is the major known driver of iTreg differentiation, IL-10 has been shown to be another key player that has been vastly described in protection from allergic diseases [39].

Studies with bee venom-specific immunotherapy have shown that tolerance to the allergen can be induced in a process that is IL-10 mediated [40]. In addition, respiratory exposure tolerance induction to OVA relied on antigen specific CD4^+^ regulatory cells that produced IL-10 [41]. Tolerance was transferrable and abrogated when IL-10 or ICOS ligand was blocked. Interestingly, those regulatory cells shared some features with effector Th2 cells: both populations expressed IL-4 and IL-10 although in different amounts. While the regulatory cells primarily release IL-10, the effectors rely on IL-4 as the main cytokine. It has been suggested that different types of effector cells, including Th2, produce IL-10 at the end of the immune response in a mechanism that is important in limiting their inflammatory behavior [42]. IL-10 producing T cells has been described able to control the late response in allergic asthma by reducing neutrophilia [43]. It has been suggested that Foxp3-negative IL-10 producing T cells can be induced following activation in presence of IL-10 and constitute a population of regulatory cells different from Foxp3^+^ Tregs that are named TR1 [44, 45]. TR1 cells have been identified in mice and humans, and there are currently clinical trials [9, 46]. There are several other lines of evidence demonstrating the crucial role of IL-10 in the prevention of airway inflammation: IL-10-deficient mice have an exacerbated allergic airways response with high levels of proinflammatory cytokines like IL-5 and IFN-*γ* in the BAL [47]. Furthermore, intranasal administration of rmIL-10, concurrently with OVA, inhibited both airway neutrophilia and eosinophilia [48]. It was also shown that allergen-specific CD4^+^CD25^+^ Tregs can suppress allergic airway disease *in vivo* through an IL-10-dependent mechanism [18]. In this study, adoptive transfer of Treg cells reduced AHR, Th2 and eosinophil recruitment into the airways, and secretion of Th2-type cytokines. The effect was IL-10 mediated, since neutralizing anti-IL-10R abrogated suppression. In addition, these effects were independent of IL-10 production by the CD4^+^CD25^+^ regulatory cells themselves [18].

Unlike TGF-*β*, IL-10 does not directly influence B-cell class switch [49]. However, it is possible that indirectly, by inhibiting the inflammatory response, IL-10 shapes the humoral outcome. Indeed, it was proposed that IL-10 may favor the ratio of IgG4/IgE ratio [50]. In fact immunotherapy studies show that Th2 responses can be suppressed by IL-10 secreting regulatory cells accompanied by an increase of circulating IgG4 [51, 52].

## 4. Different Subsets of Regulatory T Cells

Foxp3^+^ Treg cells, despite an apparent phenotypic uniformity and immunosuppressive function, can have different subtypes with distinct genetic signatures. The first major division was identified between nTreg and iTreg, where the first are enriched in Helios, a transcription factor that is primarily expressed in T-lineage cells and early precursors [53, 54]. While nTreg cells have epigenetic mechanisms that stabilize Foxp3 expression allowing them to be a stable differentiated cell lineage, TGF-*β* induced Tregs lack those mechanisms having incomplete demethylation [55]. Therefore, although iTreg cells have high levels of Foxp3, the expression of Foxp3 is less stable [55–57]. In addition, conserved noncoding DNA sequence (CNS) elements at the Foxp3 locus encode information defining the size, composition, and stability of the Treg cell population [58]. CNS3, which binds c-Rel, has a drastic effect on the frequency of Treg cells generated in the thymus. Contrary to CNS3, CNS1 has no effect on thymic generation of Treg cells but is essential for induction of iTregs [58]. CNS1 contains a TGF-*β*-NFAT response element, so these results could represent the requirement of TGF-*β* and NFAT for Treg induction in the periphery [58–60]. Although CNS2-deficient T cells can acquire Foxp3 expression, they fail to maintain Foxp3 expression on their progeny due to the failure on recruitment of Foxp3-Runx1-Cbf-*β* complexes to CNS2 after demethylation of the CNS2 CpG island [58, 61]. Interestingly CNS1 deficient mice had no lymphoproliferative disorder. However, it can be argued that these animals kept in clean facilities have a minimal exposure to foreign antigens and thus nTreg may be sufficient to maintain homeostasis in such conditions. In effector T cells, GATA-3 is a hallmark of the Th2 cells, but Treg cells can also express GATA-3, that binds both to the Th2 cell locus and to the CNS2 of Foxp3 locus [62]. In fact, there is a dramatic increase of GATA-3 binding to CNS2 compared to conventional T cells, suggesting that GATA-3 regulates CNS2 activity in Treg cells [62].

There is strong evidence that the CCR7-dependent continuous migration of DC from the lung to its draining LNs is required for the transport of inhaled Ag and thereby for the proper composition of APCs in the LN. These processes are essential to induce peripheral tolerance of T cells [63]. The costimulation with ICOS, crucial for regulatory phenotype polarization in allergy [64], promotes the downregulation of CCR7 and CD62L after activation, leading to a reduced return of activated CD4 T cells to the lymph nodes and a more efficient entry into the lungs [65]. Regulatory T cells express CCR4 and CD103 induced by antigen-driven activation in the lymph nodes. In addition, the accumulation of Tregs in the skin and lung airways is impaired in the absence of CCR4 expression [66]. Mice without CCR4 in the Treg compartment develop lymphocytic infiltration and severe inflammatory disease in the skin and lungs [66]. Some studies suggest that CCR4 has a prominent role in effector Th2 homing [67]. Despite their differences it seems both regulatory and effector T cells share the response to homing factors [68, 69].

But GATA-3 is not the only transcription factor characteristic of effector T-cell responses that can be expressed by Foxp3^+^ Treg cells. Under the influence of IFN-*γ*, Foxp3^+^ Treg cells can express the Th1-defining transcription factor T-bet [70]. T-bet expression by Foxp3^+^ Treg cells induces the expression of the chemokine receptor CXCR3, necessary for these Treg cells to accumulate at the site of type 1 inflammation. T-bet expression was thus required for the homeostasis and function of Treg cells during type-1 inflammation [70].

**Figure 1:**
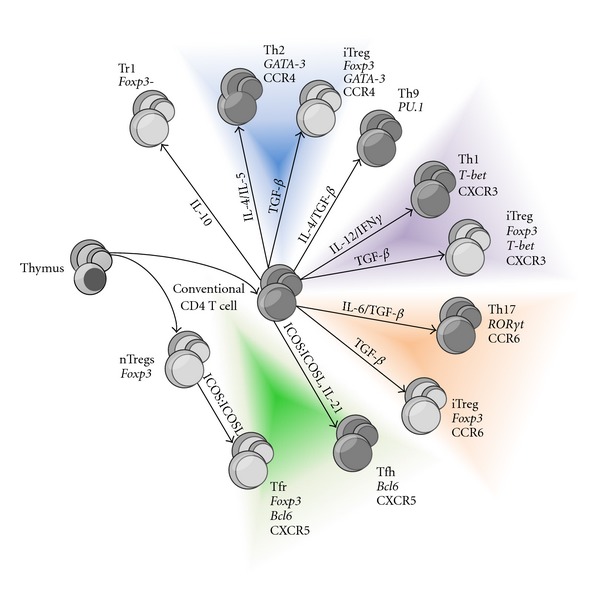
Functional specialization of effector and regulatory T cells. Different types of immune responses carry different cytokine microenvironments that can influence both effector and regulatory T-cell differentiation. In the same way effector T cells when activated in specific cytokine environment acquire specialized functions, induced regulatory cells (iTreg) can also activate the expression of different transcription factors (italics) that endow them access to different anatomic compartments on the basis of the chemokine receptors they express. Follicular regulatory cells (Tfr) represent an exception among peripherally induced Foxp3^+^ cells, as they are derived from natural regulatory cells (nTreg) that acquire Bcl-6 expression, rather than from conventional CD4 T cells.

It is likely that the regulation of different types of immune response requires the participation of specialized subsets of regulatory cells. This way, iTreg cells induced in an environment favorable to Th1 or Th2 type of immune responses require the appropriate chemokine receptors to give them access to the same locations as effector T cells (Figure 1).

Th17 cells that have been implicated in autoimmunity and allergy share with iTreg cells the need for TGF-*β* to differentiate [71]. The decision of antigen-stimulated cells to differentiate into either Th17 or iTreg depends on the cytokine balance of IL-6, IL-21, and IL-23 that relieve Foxp3-mediated inhibition of ROR*γ*t [72]. These results indicate that Foxp3 and ROR*γ*t are transcription factors that antagonize each other in the lineage differentiation. 

Another subset of T cells, the follicular T helper cells (Tfh), is mostly spatially confined to secondary lymphoid organs, more precisely to the B-cell follicles [73]. Tfh cells express high levels of the transcription factor Bcl-6, that impairs the expression and function of other transcription factors specific for other CD4 subsets: Tbet, GATA3, and ROR*γ*t, thereby regulating cytokine production by Tfh cells [74, 75]. Tfh cells differentiate under the influence of ICOS:ICOSL and IL-21 but independently of any other cytokine [76]. In addition, the characteristic anatomical distribution of Tfh cells is dependent of CXCR5 that endows access to the B-cell follicle [73, 77, 78]. We and others have recently found that also this subset of effector T cells has a specialized regulatory counterpart [69, 79, 80]. It was found that Foxp3^+^ Treg cells can be found within the B-cell follicle [81], sharing many characteristics of Tfh and Treg cells [69, 79, 80]. Importantly, Bcl-6 can be coexpressed with Foxp3 as it seems Foxp3 expression is not inhibited by Bcl-6. These follicular regulatory T cells (Tfr) are immune-suppressive and can control de magnitude of the germinal center response [69, 79, 80]. In addition, they exhibit a CTLA4^hi^GITR^hi^IL-10^hi^ phenotype that is the characteristic of activated Tregs [69, 79, 80]. However, the Tfr origin is quite distinct from the other induced Treg cells previously described. Tfr cells do not derive from the commitment of conventional CD4 T cells, but result from acquisition of “follicular” characteristics (viz. Bcl-6 expression) by natural Foxp3^+^ Treg cells [69, 79, 80]. In fact sorted Tfh cells exposed to optimal conditions to induce Foxp3 expression in conventional T cells (including TGF-*β*) resist conversion to Tfr [79]. Given the importance of the germinal center response for allergy, it is likely that Tfr cells can play an important role in regulating IgE production.

Besides conventional T cells, also natural killer T (NKT) cells are important players in defining the outcome of immune responses. Notably, invariant NKT (iNKT) cells were found able to help B-cell differentiation, germinal-center formation, affinity maturation, and immunoglobulin response that was uniquely dependent on iNKT cell-derived IL-21, although the GCs maintain a small size throughout the reaction [82, 83]. This contribution of iNKT cells for humoral responses can be added to their ability to contribute to allergic airways diseases by producing IL-4 and IL-13 [84, 85], or IL-17 [86, 87]. But iNKT cells can also have a regulatory role, namely, in preventing EAE following administration of its TCR agonist [88, 89]. We and others recently described that activation of murine or human iNKT cells in presence of TGF-*β* induces Foxp3 expression and acquisition of suppressive function [88, 90]. 

## 5. Influencing Regulatory T Cells in Allergy

The understanding of the mechanisms involved in regulatory T-cells generation and function may lead to novel strategies to restore immune tolerance where it has been lost. As TGF-*β* and IL-10 play a crucial role in tolerance induction, several studies on immune tolerance induction took advantage of environments rich in those anti-inflammatory cytokines. To our advantage the mucosa itself is an anatomical location rich in these immune mediators [91]. 

Airborne antigens can be transferred from the mother to the newborn through milk [92]. Breastfeeding-induced tolerance was found to be mediated by induced Foxp3^+^ Treg cells and dependent on TGF-*β* [92]. It has been proposed that metallomatrix proteases, derived from commensal bacteria in the gut, can facilitate the conversion of latent TGF-*β* to its active form, thus favoring iTreg differentiation [93]. In addition, CD103^+^ dendritic cells in the mucosa-draining lymph nodes have been shown effective in promoting conversion of iTregs in the gut, mediated by TGF-*β* and the synthesis of retinoic acid, a powerful inducer of Foxp3 expression [32, 94, 95]. Furthermore, vitamin D receptor deficient mice were associated with a reduction in tolerogenic CD103^+^ dendritic cells favoring the development of effector type T cells [96]. Vitamin D3 can be used to induce human and mouse naive CD4^+^ T cells to differentiate *in vitro* into regulatory cells that produced only IL-10, but no IL-5 and IFN-*γ*, and furthermore retain strong proliferative capacity [97]. Several other studies put vitamin D3 in relevance as acting directly on T cells to induce IL-10^+^ regulatory cells and also influencing levels of TGF-*β* [98–100]. These data suggest that the mucosa, in particular the gut, has several mechanisms that can favor immune tolerance. Sublingual immunotherapy (SLIT) and oral immunotherapy (OIT) are becoming more relevant as effective tolerance-inducing strategies to treat inhalant as well as food allergies [101]. 

Allergen specific immunotherapy (SIT) which comprehends SLIT, OIT, and subcutaneous immunotherapy (SCIT) has been in clinical use for around 100 years [102] and consists on the administration of increasing doses of an allergen [103]. It has been shown that both Foxp3^+^ and IL-10 positive regulatory T cells can be induced during the course of SIT protocols [104, 105]. Furthermore, allergen-specific TR1 cells, in healthy individuals, have been suggested to play a key role in preventing pathologic responses [52, 102, 106]. While the presence of IL-10 leads B cells to produce IgG4 in detriment of IgE [107, 108], TGF-*β* drives B cells to switch to IgA production [106]. Another approach to direct the organism towards a tolerant state arises from the results that suggest that reduced TCR stimulation favors the induction of a regulatory phenotype on the T cells [26, 109, 110]. Blockade of molecules involved in the immune synapse has been suggested as an approach to achieve suboptimal TCR activation [26, 110]. Blockade of CD4 was shown a robust approach to achieve Treg-mediated dominant tolerance in transplantation [111–113]. We recently showed that a nondepleting anti-CD4 monoclonal antibody can induce in mice robust, antigen-specific tolerance to house dust mite, even in presensitized animals [16]. In addition, a similar strategy was effective to prevent peanut-induced anaphylaxis in mice [114]. Costimulation blockade was also shown effective in preventing allergic sensitization in mice [115]. Based on previous studies of tolerance induction to alloantigens following costimulation blockade, it is likely the mechanism also relies on Treg cells [116, 117]. Regarding the different modalities for costimulation blockade, on one hand CTLA4Ig was shown able to greatly reduce the secretion of IL-4 but not enough to impair Th2 response [118]. On the other hand, treatment with OX40L-blocking mAbs inhibited to some extent allergic immune responses induced by TSLP in the lung and skin, preventing Th2 inflammatory cell infiltration, cytokine secretion, and IgE production in mice and nonhuman primate models of asthma [119]. 

## 6. Final Remarks

The realization that active regulatory mechanisms, such as the ones mediated by Treg cells, can prevent pathological immune responses to harmless antigens is changing the way immunotherapy is perceived. In very diverse fields of immunology, ranging from cancer immunotherapy to autoimmunity and allergy, regulatory mechanisms need to be considered when therapeutic interventions are designed to boost or dampen the immune response. The realization that different subsets of regulatory T cells exist may offer the possibility to fine tune such interventions in order to achieve optimal therapeutic benefit with limited immunosuppressive consequences in unrelated immune responses.

At a time when therapeutic interventions rely increasingly on potentially immunogenic drugs, such as recombinant proteins to correct genetic diseases or monoclonal antibodies, where even the human antibodies can be immunogenic due to their unique idiotypes [120, 121], the issue of tolerance induction to nonselfantigens will not be restricted to allergy and transplantation, but a growing concern for drug efficacy.

## References

[1] Takahashi T, Kuniyasu Y, Toda M (1998). Immunologic self-tolerance maintained by CD25^+^CD4^+^ naturally anergic and suppressive T cells: induction of autoimmune disease by breaking their anergic/suppressive state. *International Immunology*.

[2] Douwes J, Brooks C, van Dalen C, Pearce N (2011). Importance of allergy in asthma: an epidemiologic perspective. *Current Allergy and Asthma Reports*.

[3] Rudensky AY (2011). Regulatory T cells and *Foxp3*. *Immunological Reviews*.

[4] Sakaguchi S, Wing K, Miyara M (2007). Regulatory T cells—a brief history and perspective. *European Journal of Immunology*.

[5] Valencia X, Lipsky PE (2007). CD4^+^CD25^+^
*Foxp3*
^+^ regulatory T cells in autoimmune diseases. *Nature Clinical Practice Rheumatology*.

[6] Ling EM, Smith T, Nguyen XD (2004). Relation of CD4^+^CD25^+^ regulatory T-cell suppression of allergen-driven T-cell activation to atopic status and expression of allergic disease. *The Lancet*.

[7] Waldmann H, Chen TC, Graca L (2006). Regulatory T cells in transplantation. *Seminars in Immunology*.

[8] Graca L, Le Moine A, Cobbold SP, Waldmann H (2003). Dominant transplantation tolerance: opinion. *Current Opinion in Immunology*.

[9] Wood KJ, Bushell A, Hester J (2012). Regulatory immune cells in transplantation. *Nature Reviews Immunology*.

[10] Cottrez F, Hurst SD, Coffman RL, Groux H (2000). T regulatory cells 1 inhibit a Th2-specific response *in vivo*. *Journal of Immunology*.

[11] Navarro S, Cossalter G, Chiavaroli C (2011). The oral administration of bacterial extracts prevents asthma via the recruitment of regulatory T cells to the airways. *Mucosal Immunology*.

[12] Suto A, Nakajima H, Kagami SI, Suzuki K, Saito Y, Iwamoto I (2001). Role of CD4^+^ CD25^+^ regulatory T cells in T helper 2 cell-mediated allergic inflammation in the airways. *American Journal of Respiratory and Critical Care Medicine*.

[13] Bennett CL, Christie J, Ramsdell F (2001). The immune dysregulation, polyendocrinopathy, enteropathy, X-linked syndrome (IPEX) is caused by mutations of *Foxp3*. *Nature Genetics*.

[14] Curotto de Lafaille MA, Lafaille JJ (2009). Natural and adaptive *Foxp3*
^+^ regulatory T cells: more of the same or a division of labor?. *Immunity*.

[15] Chen W, Jin W, Hardegen N (2003). Conversion of peripheral CD4^+^CD25^−^ naive T cells to CD4^+^CD25^+^ regulatory T cells by TGF-*β* induction of transcription factor *Foxp3*. *Journal of Experimental Medicine*.

[16] Agua-Doce A, Graca L (2011). Prevention of house dust mite induced allergic airways disease in mice through immune tolerance. *PLoS ONE*.

[17] Strickland DH, Stumbles PA, Zosky GR (2006). Reversal of airway hyperresponsiveness by induction of airway mucosal CD4^+^CD25^+^ regulatory T cells. *Journal of Experimental Medicine*.

[18] Kearley J, Barker JE, Robinson DS, Lloyd CM (2005). Resolution of airway inflammation and hyperreactivity after *in vivo* transfer of CD4^+^CD25^+^ regulatory T cells is interleukin 10 dependent. *Journal of Experimental Medicine*.

[19] Lewkowich IP, Herman NS, Schleifer KW (2005). CD4^+^CD25^+^ T cells protect against experimentally induced asthma and alter pulmonary dendritic cell phenotype and function. *Journal of Experimental Medicine*.

[20] Scherf W, Burdach S, Hansen G (2005). Reduced expression of transforming growth factor *β*1 exacerbates pathology in an experimental asthma model. *European Journal of Immunology*.

[21] Nakao A, Miike S, Hatano M (2000). Blockade of transforming growth factor *β*/Smad signaling in T cells by overexpression of Smad7 enhances antigen-induced airway inflammation and airway reactivity. *Journal of Experimental Medicine*.

[22] Hansen G, McIntire JJ, Yeung VP (2000). CD4^+^ T helper cells engineered to produce latent TGF-*β*1 reverse allergen-induced airway hyperreactivity and inflammation. *Journal of Clinical Investigation*.

[23] Joetham A, Takada K, Taube C (2007). Naturally occurring lung CD4^+^CD25^+^ T cell regulation of airway allergic responses depends on IL-10 induction of TGF- *β*1. *Journal of Immunology*.

[24] Kretschmer K, Apostolou I, Hawiger D, Khazaie K, Nussenzweig MC, von Boehmer H (2005). Inducing and expanding regulatory T cell populations by foreign antigen. *Nature Immunology*.

[25] Ostroukhova M, Seguin-Devaux C, Oriss TB (2004). Tolerance induced by inhaled antigen involves CD4^+^ T cells expressing membrane-bound TGF-*β* and FOXP3. *Journal of Clinical Investigation*.

[26] Oliveira VG, Caridade M, Paiva RS, Demengeot J, Graca L (2011). Sub-optimal CD4^+^ T-cell activation triggers autonomous TGF-*β*-dependent conversion to *Foxp3*+ regulatory T cells. *European Journal of Immunology*.

[27] Gorelik L, Fields PE, Flavell RA (2000). Cutting edge: TGF-*β* inhibits Th type 2 development through inhibition of GATA-3 expression. *Journal of Immunology*.

[28] Heath VL, Murphy EE, Crain C, Tomlinson MG, O’Garra A (2000). TGF-*β*1 down-regulates Th2 development and results in decreased IL-4-induced STAT6 activation and GATA-3 expression. *European Journal of Immunology*.

[29] Chen CH, Seguin-Devaux C, Burke NA (2003). Transforming growth factor *β* blocks Tec kinase phosphorylation, Ca^2+^ influx, and NFATc translocation causing inhibition of T cell differentiation. *Journal of Experimental Medicine*.

[30] van Vlasselaer P, Punnonen J, de Vries JE (1992). Transforming growth factor-*β* directs IgA switching in human B cells. *Journal of Immunology*.

[31] Gereke M, Jung S, Buer J, Bruder D (2009). Alveolar type II epithelial cells present antigen to CD4^+^ T cells and induce *Foxp3*
^+^ regulatory T cells. *American Journal of Respiratory and Critical Care Medicine*.

[32] Mucida D, Park Y, Kim G (2007). Reciprocal TH17 and regulatory T cell differentiation mediated by retinoic acid. *Science*.

[33] Goswami S, Angkasekwinai P, Shan M (2009). Divergent functions for airway epithelial matrix metalloproteinase 7 and retinoic acid in experimental asthma. *Nature Immunology*.

[34] Takaki H, Ichiyama K, Koga K (2008). STAT6 inhibits TGF-*β*1-mediated *Foxp3* induction through direct binding to the *Foxp3* promoter, which is reverted by retinoic acid receptor. *The Journal of Biological Chemistry*.

[35] Veldhoen M, Uyttenhove C, van Snick J (2008). Transforming growth factor-*β* “reprograms” the differentiation of T helper 2 cells and promotes an interleukin 9-producing subset. *Nature Immunology*.

[36] Korn T, Mitsdoerffer M, Croxford AL (2008). IL-6 controls Th17 immunity *in vivo* by inhibiting the conversion of conventional T cells into *Foxp3*
^+^ regulatory T cells. *Proceedings of the National Academy of Sciences of the United States of America*.

[37] Veldhoen M, Hocking RJ, Flavell RA, Stockinger B (2006). Signals mediated by transforming growth factor-*β* initiate autoimmune encephalomyelitis, but chronic inflammation is needed to sustain disease. *Nature Immunology*.

[38] Makinde T, Murphy RF, Agrawal DK (2007). The regulatory role of TGF-*β* in airway remodeling in asthma. *Immunology and Cell Biology*.

[39] Rubtsov YP, Rasmussen JP, Chi EY (2008). Regulatory T cell-derived interleukin-10 limits inflammation at environmental interfaces. *Immunity*.

[40] Akdis CA, Blesken T, Akdis M, Wüthrich B, Blaser K (1998). Role of interleukin 10 in specific immunotherapy. *Journal of Clinical Investigation*.

[41] Akbari O, Freeman GJ, Meyer EH (2002). Antigen-specific regulatory T cells develop via the ICOS-ICOS-ligand pathway and inhibit allergen-induced airway hyperreactivity. *Nature Medicine*.

[42] O’Garra A, Barrat FJ, Castro AG, Vicari A, Hawrylowicz C (2008). Strategies for use of IL-10 or its antagonists in human disease. *Immunological Reviews*.

[43] Nabe T, Ikedo A, Hosokawa F (2012). Regulatory role of antigen-induced interleukin-10, produced by CD4^+^ T cells, in airway neutrophilia in a murine model for asthma. *European Journal of Pharmacology*.

[44] Lloyd CM, Hawrylowicz CM (2009). Regulatory T cells in asthma. *Immunity*.

[45] Levings MK, Roncarolo MG (2000). T-regulatory 1 cells: a novel subset of CD4^+^ T cells with immunoregulatory properties. *Journal of Allergy and Clinical Immunology*.

[46] Battaglia M, Roncarolo MG (2011). Immune intervention with T regulatory cells: past lessons and future perspectives for type 1 diabetes. *Seminars in Immunology*.

[47] Grünig G, Corry DB, Leach MW, Seymour BWP, Kurup VP, Rennick DM (1997). Interleukin-10 is natural suppressor of cytokine production and inflammation in a murine model of allergic bronchopulmonary aspergillosis. *Journal of Experimental Medicine*.

[48] Zuany-Amorim C, Haile S, Leduc D (1995). Interleukin-10 inhibits antigen-induced cellular recruitment into the airways of sensitized mice. *Journal of Clinical Investigation*.

[49] Hummelshoj L, Ryder LP, Poulsen LK (2006). The role of the interleukin-10 subfamily members in immunoglobulin production by human B cells. *Scandinavian Journal of Immunology*.

[50] Till SJ, Francis JN, Nouri-Aria K, Durham SR (2004). Mechanisms of immunotherapy. *Journal of Allergy and Clinical Immunology*.

[51] Akdis M, Akdis CA (2007). Mechanisms of allergen-specific immunotherapy. *Journal of Allergy and Clinical Immunology*.

[52] Meiler F, Zumkehr J, Klunker S, Rückert B, Akdis CA, Akdis M (2008). *In vivo* switch to IL-10-secreting T regulatory cells in high dose allergen exposure. *Journal of Experimental Medicine*.

[53] Thornton AM, Korty PE, Tran DQ (2010). Expression of Helios, an Ikaros transcription factor family member, differentiates thymic-derived from peripherally induced *Foxp3*
^+^ T regulatory cells. *Journal of Immunology*.

[54] Haribhai D, Lin W, Edwards B (2009). A central role for induced regulatory T cells in tolerance induction in experimental colitis. *Journal of Immunology*.

[55] Floess S, Freyer J, Siewert C (2007). Epigenetic control of the *Foxp3* locus in regulatory T cells. *PLoS Biology*.

[56] Polansky JK, Kretschmer K, Freyer J (2008). DNA methylation controls *Foxp3* gene expression. *European Journal of Immunology*.

[57] Miyao T, Floess S, Setoguchi R (2012). Plasticity of *Foxp3*
^+^ T cells reflects promiscuous *Foxp3* expression in conventional T cells but not reprogramming of regulatory T cells. *Immunity*.

[58] Zheng Y, Josefowicz S, Chaudhry A, Peng XP, Forbush K, Rudensky AY (2010). Role of conserved non-coding DNA elements in the *Foxp3* gene in regulatory T-cell fate. *Nature*.

[59] Tone Y, Furuuchi K, Kojima Y, Tykocinski ML, Greene MI, Tone M (2008). Smad3 and NFAT cooperate to induce *Foxp3* expression through its enhancer. *Nature Immunology*.

[60] Liu Y, Zhang P, Li J, Kulkarni AB, Perruche S, Chen W (2008). A critical function for TGF-*β* signaling in the development of natural CD4^+^CD25^+^
*Foxp3*
^+^ regulatory T cells. *Nature Immunology*.

[61] Kitoh A, Ono M, Naoe Y (2009). Indispensable role of the Runx1-Cbf*β* transcription complex for *in vivo*-suppressive function of *Foxp3*
^+^ regulatory T cells. *Immunity*.

[62] Wang Y, Su M, Wan Y (2011). An essential role of the transcription factor GATA-3 for the function of regulatory T cells. *Immunity*.

[63] Hintzen G, Ohl L, Del Rio ML (2006). Induction of tolerance to innocuous inhaled antigen relies on a CCR7-dependent dendritic cell-mediated antigen transport to the bronchial lymph node. *Journal of Immunology*.

[64] Akbari O, Stock P, Singh AK (2010). PD-L1 and PD-L2 modulate airway inflammation and iNKT-cell-dependent airway hyperreactivity in opposing directions. *Mucosal Immunology*.

[65] Moore TV, Clay BS, Cannon JL, Histed A, Shilling RA, Sperling AI (2011). Inducible costimulator controls migration of T cells to the lungs via down-regulation of CCR7 and CD62L. *American Journal of Respiratory Cell and Molecular Biology*.

[66] Sather BD, Treuting P, Perdue N (2007). Altering the distribution of *Foxp3*
^+^ regulatory T cells results in tissue-specific inflammatory disease. *Journal of Experimental Medicine*.

[67] Imai T, Nagira M, Takagi S (1999). Selective recruitment of CCR4-bearing Th2 cells toward antigen-presenting cells by the CC chemokines thymus and activation-regulated chemokine and macrophage-derived chemokine. *International Immunology*.

[68] Hall BM, Verma ND, Tran GT, Hodgkinson SJ (2011). Distinct regulatory CD4 ^+^ T cell subsets; differences between naïve and antigen specific T regulatory cells. *Current Opinion in Immunology*.

[69] Linterman MA, Pierson W, Lee SK (2011). *Foxp3*
^+^ follicular regulatory T cells control the germinal center response. *Nature Medicine*.

[70] Koch MA, Tucker-Heard G, Perdue NR, Killebrew JR, Urdahl KB, Campbell DJ (2009). The transcription factor T-bet controls regulatory T cell homeostasis and function during type 1 inflammation. *Nature Immunology*.

[71] Bettelli E, Carrier Y, Gao W (2006). Reciprocal developmental pathways for the generation of pathogenic effector TH17 and regulatory T cells. *Nature*.

[72] Zhou L, Lopes JE, Chong MMW (2008). TGF-*β*-induced *Foxp3* inhibits TH17 cell differentiation by antagonizing ROR*γ*t function. *Nature*.

[73] Haynes NM, Allen CDC, Lesley R, Ansel KM, Killeen N, Cyster JG (2007). Role of CXCR5 and CCR7 in follicular Th cell positioning and appearance of a programmed cell death gene-1High germinal center-associated subpopulation. *Journal of Immunology*.

[74] Yu D, Rao S, Tsai LM (2009). The transcriptional repressor Bcl-6 directs T follicular helper cell lineage commitment. *Immunity*.

[75] Nurieva RI, Chung Y, Martinez GJ (2009). Bcl6 mediates the development of T follicular helper cells. *Science*.

[76] Nurieva RI, Chung Y, Hwang D (2008). Generation of T follicular helper cells is mediated by interleukin-21 but independent of T helper 1, 2, or 17 cell lineages. *Immunity*.

[77] Breitfeld D, Ohl L, Kremmer E (2000). Follicular B helper T cells express CXC chemokine receptor 5, localize to B cell follicles, and support immunoglobulin production. *Journal of Experimental Medicine*.

[78] Schaerli P, Willimann K, Lang AB, Lipp M, Loetscher P, Moser B (2000). CXC chemokine receptor 5 expression defines follicular homing T cells with B cell helper function. *Journal of Experimental Medicine*.

[79] Wollenberg I, Agua-Doce A, Hernández A (2011). Regulation of the germinal center reaction by *Foxp3*
^+^ follicular regulatory T cells. *Journal of Immunology*.

[80] Chung Y, Tanaka S, Chu F (2011). Follicular regulatory T cells expressing *Foxp3* and Bcl-6 suppress germinal center reactions. *Nature Medicine*.

[81] Lim HW, Hillsamer P, Kim CH (2004). Regulatory T cells can migrate to follicles upon T cell activation and suppress GC-Th cells and GC-Th cell-driven B cell responses. *Journal of Clinical Investigation*.

[82] Chang PP, Barral P, Fitch J (2012). Identification of Bcl-6-dependent follicular helper NKT cells that provide cognate help for B cell responses. *Nature Immunology*.

[83] King IL, Fortier A, Tighe M (2012). Invariant natural killer T cells direct B cell responses to cognate lipid antigen in an IL-21-dependent manner. *Nature Immunology*.

[84] Akbari O, Stock P, Meyer E (2003). Essential role of NKT cells producing IL-4 and IL-13 in the development of allergen-induced airway hyperreactivity. *Nature Medicine*.

[85] Lisbonne M, Diem S, de Castro Keller A (2003). Cutting edge: invariant V*α*14 NKT cells are required for allergen-induced airway inflammation and hyperreactivity in an experimental asthma model. *Journal of Immunology*.

[86] Michel ML, Keller AC, Paget C (2007). Identification of an IL-17-producing NK1.1neg iNKT cell population involved in airway neutrophilia. *Journal of Experimental Medicine*.

[87] Coquet JM, Chakravarti S, Kyparissoudis K (2008). Diverse cytokine production by NKT cell subsets and identification of an IL-17-producing CD4^−^NK1.1^−^ NKT cell population. *Proceedings of the National Academy of Sciences of the United States of America*.

[88] Monteiro M, Almeida CF, Caridade M (2010). Identification of regulatory *Foxp3*
^+^ invariant NKT cells induced by TGF-*β*. *Journal of Immunology*.

[89] Mars LT, Araujo L, Kerschen P (2009). Invariant NKT cells inhibit development of the Th17 lineage. *Proceedings of the National Academy of Sciences of the United States of America*.

[90] Moreira-Teixeira L, Resende M, Devergne O (2012). Rapamycin combined with TGF-*β* converts human invariant NKT Cells into suppressive *Foxp3*
^+^ regulatory cells. *Journal of Immunology*.

[91] Konkel JE, Chen W (2011). Balancing acts: the role of TGF-*β* in the mucosal immune system. *Trends in Molecular Medicine*.

[92] Verhasselt V, Milcent V, Cazareth J (2008). Breast milk-mediated transfer of an antigen induces tolerance and protection from allergic asthma. *Nature Medicine*.

[93] Atarashi K, Tanoue T, Shima T (2011). Induction of colonic regulatory T cells by indigenous *Clostridium* species. *Science*.

[94] Benson MJ, Pino-Lagos K, Rosemblatt M, Noelle RJ (2007). All-trans retinoic acid mediates enhanced T reg cell growth, differentiation, and gut homing in the face of high levels of co-stimulation. *Journal of Experimental Medicine*.

[95] Coombes JL, Siddiqui KRR, Arancibia-Cárcamo CV (2007). A functionally specialized population of mucosal CD103^+^ DCs induces *Foxp3*
^+^ regulatory T cells via a TGF-*β* -and retinoic acid-dependent mechanism. *Journal of Experimental Medicine*.

[96] Bruce D, Yu S, Ooi JH, Cantorna MT (2011). Converging pathways lead to overproduction of IL-17 in the absence of vitamin D signaling. *International Immunology*.

[97] Barrat FJ, Cua DJ, Boonstra A (2002). *In vitro* generation of interleukin 10-producing regulatory CD4^+^ T cells is induced by immunosuppressive drugs and inhibited by T helper type 1 (Th1)- and Th2-inducing cytokines. *Journal of Experimental Medicine*.

[98] Penna G, Roncari A, Amuchastegui S (2005). Expression of the inhibitory receptor ILT3 on dendritic cells is dispensable for induction of CD4^+^
*Foxp3*
^+^ regulatory T cells by 1,25-dihydroxyvitamin D3. *Blood*.

[99] Urry Z, Xystrakis E, Richards DF (2009). Ligation of TLR9 induced on human IL-10-secreting Tregs by 1*α*,25-dihydroxyvitamin D3 abrogates regulatory function. *Journal of Clinical Investigation*.

[100] Mahon BD, Wittke A, Weaver V, Cantorna MT (2003). The targets of vitamin D depend on the differentiation and activation status of CD4 positive T cells. *Journal of Cellular Biochemistry*.

[101] Noh G, Lee JH (2012). Oral tolerance induction for human food allergy. *Inflammation and Allergy—Drug Targets*.

[102] Akdis CA, Akdis M (2011). Mechanisms of allergen-specific immunotherapy. *Journal of Allergy and Clinical Immunology*.

[103] Fujita H, Soyka MB, Akdis M, Akdis CA (2012). Mechanisms of allergen-specific immunotherapy. *Clinical and Translational Allergy*.

[104] Akdis M, Blaser K, Akdis CA (2005). T regulatory cells in allergy: novel concepts in the pathogenesis, prevention, and treatment of allergic diseases. *Journal of Allergy and Clinical Immunology*.

[105] Palomares O, Yaman G, Azkur AK, Akkoc T, Akdis M, Akdis CA (2010). Role of Treg in immune regulation of allergic diseases. *European Journal of Immunology*.

[106] Jutel M, Akdis M, Budak F (2003). IL-10 and TGF-*β* cooperate in the regulatory T cell response to mucosal allergens in normal immunity and specific immunotherapy. *European Journal of Immunology*.

[107] Carballido JM, Carballido-Perrig N, Kagi MK (1993). T cell epitope specificity in human allergic and nonallergic subjects to bee venom phospholipase A2. *Journal of Immunology*.

[108] Platts-Mills TAE, Vaughan JW, Blumenthal K, Woodfolk JA, Sporik RB (2001). Decreased prevalence of asthma among children with high exposure to cat allergen: relevance of the modified Th2 response. *Mediators of Inflammation*.

[109] Kim JM, Rudensky A (2006). The role of the transcription factor *Foxp3* in the development of regulatory T cells. *Immunological Reviews*.

[110] Graca L, Chen TC, Moine AL, Cobbold SP, Howie D, Waldmann H (2005). Dominant tolerance: activation thresholds for peripheral generation of regulatory T cells. *Trends in Immunology*.

[111] Lin CY, Graca L, Cobbold SP, Waldmann H (2002). Dominant transplantation tolerance impairs CD8^+^ T cell function but not expansion. *Nature Immunology*.

[112] Graca L, Le Moine A, Lin CY, Fairchild PJ, Cobbold SP, Waldmann H (2004). Donor-specific transplantation tolerance: the paradoxical behavior of CD4^+^CD25^+^ T cells. *Proceedings of the National Academy of Sciences of the United States of America*.

[113] Qin S, Cobbold SP, Pope H (1993). ‘Infectious’ transplantation tolerance. *Science*.

[114] Duarte J, Caridade M, Graca L (2011). CD4-blockade can induce protection from peanut-induced anaphylaxis. *Frontiers in Immunology*.

[115] Linhart B, Bigenzahn S, Hartl A (2007). Costimulation blockade inhibits allergic sensitization but does not affect established allergy in a murine model of grass pollen allergy. *Journal of Immunology*.

[116] Taylor PA, Friedman TM, Korngold R, Noelle RJ, Blazar BR (2002). Tolerance induction of alloreactive T cells via *ex vivo* blockade of the CD40:CD40L costimulatory pathway results in the generation of a potent immune regulatory cell. *Blood*.

[117] Graca L, Honey K, Adams E, Cobbold SP, Waldmann H (2000). Cutting edge: anti-CD154 therapeutic antibodies induce infectious transplantation tolerance. *Journal of Immunology*.

[118] Tang A, Judge TA, Nickoloff BJ, Turka LA (1996). Suppression of murine allergic contact dermatitis by CTLA4Ig: tolerance induction of Th2 responses requires additional blockade of CD40-ligand. *Journal of Immunology*.

[119] Seshasayee D, Lee WP, Zhou M (2007). *In vivo* blockade of OX40 ligand inhibits thymic stromal lymphopoietin driven atopic inflammation. *Journal of Clinical Investigation*.

[120] Green D (2011). Factor VIII inhibitors: a 50-year perspective. *Haemophilia*.

[121] Emi Aikawa N, de Carvalho JF, Artur Almeida Silva C, Bonfá E (2010). Immunogenicity of anti-TNF-*α* agents in autoimmune diseases. *Clinical Reviews in Allergy and Immunology*.

